# Is a less-intensive follow-up for colon cancer in early stages reasonable?

**DOI:** 10.1007/s00384-023-04350-8

**Published:** 2023-03-04

**Authors:** Katharina Esswein, Marco Volpi, Marijana Ninkovic, Veronika Kröpfl, Elisabeth Gasser, Andreas Lorenz, Lea Stecher, Reinhold Kafka-Ritsch, Stefan Schneeberger, Dietmar Öfner, Alexander Perathoner

**Affiliations:** Department of Visceral, Transplant and Thoracic Surgery, Center of Operative Medicine, Medical University of Innsbruck, Innsbruck, Austria

**Keywords:** Surveillance, Follow-up, Colon cancer, Resection

## Abstract

**Purpose:**

Patients with colon cancer are usually included in an intensive 5-year surveillance protocol after curative resection, independent of the tumor stage, though early stages have a considerably lower risk of recurrence. The aim of this study was to analyze the adherence to an intensive follow-up and the risk of recurrence in patients with colon cancer in UICC stages I and II.

**Methods:**

In this retrospective study, we assessed patients who underwent resection for colon cancer in UICC stages I and II between 2007 and 2016. Data were collected on demographics, tumor stages, therapy, surveillance, recurrent disease, and oncological outcome.

**Results:**

Of the 232 included patients, 43.5% (*n* = 101) reached the 5-year follow-up disease-free. Seven (7.5%) patients in stage UICC I and sixteen (11.5%) in UICC II had a recurrence, with the highest risk in patients with pT4 (26.3%). A metachronous colon cancer was detected in four patients (1.7%). The therapy of recurrence was intended to be curative in 57.1% (*n* = 4) of UICC stage I and in 43.8% (*n* = 7) of UICC stage II, but only in one of seven patients over 80 years. 44.8% (*n* = 104) of the patients were lost to follow-up.

**Conclusion:**

A postoperative surveillance in patients with colon cancer is important and recommended as a recurrent disease can be treated successfully in many patients. However, we suggest that a less intensive surveillance protocol is reasonable for patients with colon cancer in early tumor stages, especially in UICC stage I, as the risk of recurrent disease is low. With elderly and/or frail patients in a reduced general condition, who will not endure further specific therapy in case of a recurrence, the performance of the surveillance should be discussed: we recommend a significant reduction or even renunciation.

## Introduction

Colorectal cancer is the third most common type of cancer and the second leading cause of cancer death worldwide [1]. For colon cancer UICC (Union internationale contre le cancer) stage I, the 5-year survival rate is over 90% [2]. The standard treatment in UICC stages I and II is the surgical resection of the primary tumor. The curative therapy in UICC stage III includes adjuvant chemotherapy next to the resection of the tumor. Even in the UICC stage IV with metastases or peritoneal carcinomatosis, a cure may be achieved with multimodal treatment. A surveillance protocol usually follows the surgical resection of the primary tumor to detect a recurrent disease or metachronous colorectal cancer at an early stage. About 80% of the recurrence occur within the first 3 years and further 15% between the 3rd and 5th year [3]. Recurrent disease may occur as locoregional recurrence or distant metastases (mainly in the liver and lungs). Additionally, the risk of metachronous colorectal cancer in the remaining colon or rectum is higher, with about 0.35% per year, compared to patients without colorectal cancer [4].

Major international cancer societies, such as ESMO (European Society for Medical Oncology), recommend an intensive 5-year follow-up for all patients, independent of tumor stage. The surveillance consists of regular physical examination, carcinoembryonic antigen (CEA) testing, computed tomography (CT), and endoscopy in time intervals of 3 to 6 months [3]. This rather intensive follow-up is common practice although based on limited evidence. Several studies could not show that an intensive follow-up, compared to less frequent follow-up examinations, results in better survival [5–7]. Most studies are based on the data of patients with a tumor in UICC stages II or III. Presumably, patients in early UICC stages may not benefit from these intensive follow-up protocols because of the low recurrence rate. The risk of recurrence is about 10% in early-stage disease (UICC stages I and IIA), compared to over 30% in advanced-stage disease (UICC stages IIB and III) [8]. Furthermore, for elderly patients, the effort of an intensive follow-up must be considered (e.g., frequent visits and mechanical bowel preparation). Moreover, even in the case of early detection of a recurrence, often no cancer-specific therapy is started due to their bad clinical condition or refusal in elderly patients. Thus, the detection of recurrent disease has no clinical consequence for these patients.

This study aims to analyze the efficacy of an intensive follow-up in patients with colon cancer in UICC stages I and II regarding adherence and oncological outcome.

## Methods

**Fig. 1:**
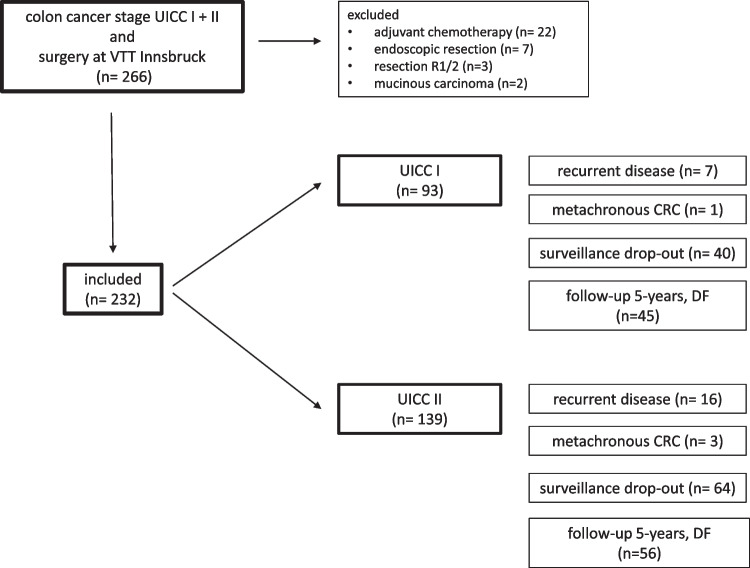
Flowchart of the included and excluded patients. VTT, Visceral, Transplant, and Thoracic Surgery; UICC, Union internationale contre le cancer; CRC, colorectal cancer; DF, disease free

All patients over the age of 18 with colon cancer in UICC stages I and II who underwent a resection of the tumor at the Medical University of Innsbruck, Department of Visceral, Transplant, and Thoracic Surgery between 2007 and 2016 were evaluated for this retrospective study. Patients with rectal cancer were excluded because of the rather complex multimodal treatment (e.g., neoadjuvant radiochemotherapy, ostomy creation, and reversal) and the higher risk of local recurrence, to create a patient collective as homogenous as possible. Patients who received adjuvant chemotherapy (recommended by the interdisciplinary tumor-board despite UICC stages I or II due to additional risk factors such as bowel perforation, high grading 3/4, positive vascular-, lymphangio-, or perineural-status) were excluded. Further exclusion criteria were endoscopic resection, R1- or R2- resection, and mucinous carcinoma. Figure 1 shows the flowchart of included and excluded patients.

The surveillance time was 5 years, according to the follow-up protocol at the Medical University of Innsbruck, Department of Visceral, Transplant, and Thoracic Surgery, based on the recommendations of NCCN (National Comprehensive Cancer Network), ESMO (European Society for Medical Oncology), and ACO ASSO (Austrian Society for Surgical Oncology) (Table 1).

**Table 1:** The surveillance protocol at the Medical University of Innsbruck, Department of Visceral, Transplant, and Thoracic Surgery based on the recommendations of NCCN (National Comprehensive Cancer Network), ESMO (European Society for Medical Oncology), and ACO ASSO (Austrian Society for Surgical Oncology)

	**1st year**				**2nd year**				**3rd year**				**4th year**				**5th year**			
**Months**	3	6	9	12	15	18	21	24	27	30	33	36	39	42	45	48	51	54	57	60
**CEA**	x	x	x	x	x	x	x	x	-	x	-	x	-	x	-	x	-	x	-	x
**Clinical examination**	x	x	x	x	x	x	x	x	-	x	-	x	-	x	-	x	-	x	-	x
**Colonoscopy**	(x^a^)	-	-	x	-	-	-	-	-	-	-	-	-	-	-	X^b^	-	-	-	-
**CT scan**	-	-	-	x	-	-	-	x	-	-	-	x	-	-	-	x	-	-	-	x

In case of curative treatment of distant metastases, CT scans are repeated in 6-monthly intervals during the first 2 years, and the surveillance period is prolonged for further 5 years

*CEA* carcinoembryonic antigen, *CT* computed tomography

^a^If not performed preoperatively

^b^If normal findings are to be repeated regularly according to national screening protocols

Data were collected from medical reports, operative reports, anesthesia protocols, and histopathological findings, by using electronic health records (Klinisches Informationssystem, KIS, Powerchart, Cerner). Demographic variables were age and gender. Clinical variables included the physical status classification system ASA (American Society of Anesthesiologists), surgical resection, histopathology evaluation, and the TNM classification. Outcome variables included surveillance drop-out, 5-year disease-free survival (DFS), and 5-year overall survival (OS). Recurrence was defined as clear radiological or endoscopic suspicion with or without histological proof. Tumor recurrence was divided into local recurrence and metastases (e.g., liver or lung metastases). Additionally, metachronous colorectal cancer was documented. Finally, the therapeutic approach of recurrence was analyzed.

Statistical analyses were performed with the software SPSS (IBM SPSS Statistics 20; International Business Machines Corporation; Armonk, New York, USA). The Kaplan–Meier method was used to calculate the DFS and OS.

The local ethics committee approved the study (Votum 1437/2021).

## Results

In total *n* = 232 patients were included, and the patient’s median (range) age was 73 (35–94) years with 47.4% female patients. The cancer stage distribution was 40.1% UICC I and 59.9% UICC II. Table 2 shows baseline information of the included patients and tumor characteristics.

**Table 2:** Baseline information of the included patients and tumor characteristics. *UICC*, Union internationale contre le cancer; *ASA*, American Society of Anesthesiologists

**Parameter**	**Number of patients**
**Gender**	
**Female**	110 (47.4%)
**Male**	122 (52.6%)
**ASA**	
**I**	15 (6.5%)
**II**	106 (45.7%)
**III**	87 (37.5%)
**IV**	6 (2.6%)
**Not documented**	18 (7.8%)
**UICC**	
**I**	93 (40.1%)
**II**	139 (59.9%)
**pT**	
**1**	44 (19.0%)
**2**	49 (21.1%)
**3**	120 (51.7%)
**4**	19 (8.2%)
**Tumor location**	
**Caecum**	47 (20.3%)
**Ascending colon**	61 (26.3%)
**Right flexure**	15 (6.5%)
**Transverse colon**	18 (7.8%)
**Left flexure**	7 (3.0%)
**Descending colon**	15 (6.5%)
**Sigmoid colon**	69 (29.7%)
**Prior tumor disease**	
**Rectal cancer**	5 (2.2%)
**Lung cancer**	5 (2.2%)
**Melanoma**	3 (1.3%)
**Bladder cancer**	3 (1.3%)
**Renal cancer**	2 (0.9%)
**Prostate cancer**	2 (0.9%)
**Breast cancer**	2 (0.9%)
**Vulvar carcinoma**	1 (0.4%)
**Anal cancer**	1 (0.4%)
**Esophageal cancer**	1 (0.4%)
**Thyroid cancer**	1 (0.4%)
**Laryngeal cancer**	1 (0.4%)
**Rhabdomyosarcoma**	1 (0.4%)
**Leukemia**	1 (0.4%)

Table 3 shows the corresponding oncological surgical resections. Five patients had a subtotal colectomy as a consequence of additional multiple polyps in various locations besides the colon cancer. A proctocolectomy was performed on one patient with a tumor and ulcerative colitis.

**Table 3:** The different oncological surgical resections

**Surgical resection**	**Recurrence *n* = 23**	**No recurrence *n* = 209**
**Right hemicolectomy**	14 (60.9%)	108 (51.7%)
**Resection of the transverse colon**	0	7 (3.3%)
**Left hemicolectomy**	2 (8.7%)	20 (9.5%)
**Sigmoid resection**	7 (30.5%)	57 (27.3%)
**Subtotal- and proctocolectomy**	0	6 (2.9%)

Apart from the standard oncological resections, five patients had an oncologically limited resection regarding lymph node dissection due to their high age and poor general condition. In another six patients, the preoperative findings showed benign polyps, and therefore patients underwent a limited resection (ileocecal resection, coecum wedge resection, segment resection, local full-thickness resection). Although the histological examination revealed malignancy, no second laparotomy to perform the usual standard oncological procedure was undertaken after carefully considering individual risks and benefits of a further operation. Six patients refused adjuvant chemotherapy, which was advised as a consequence of risk factors such as perforation, tumor differentiation grade (G3), or marginal resection.

### Follow-up

Of the 232 included patients, 43.5% (*n* = 101) reached the 5-year follow-up disease-free, and 44.8% (n = 104) were lost to follow-up after a median time of 11 months (range 0–57). The main reason for the high drop-out rate is unknown (*n* = 64), followed by death (*n* = 17), advanced age or bad general condition (*n* = 15), follow-up in other hospital (*n* = 7), and denial (*n* = 1). Death was associated with colon cancer in four of the seventeen cases (23.5%). Regarding the eleven patients with limited resection, five reached the 5-year follow-up without a recurrence, four left the follow-up after 21–36 months, and two died directly after the surgery. The overall survival (OS) after 5 years was for all 82.3%, for UICC I 87.1%, and for UICC II 79.1% (*p* = 0.082).

### Recurrent disease

**Fig. 2:**
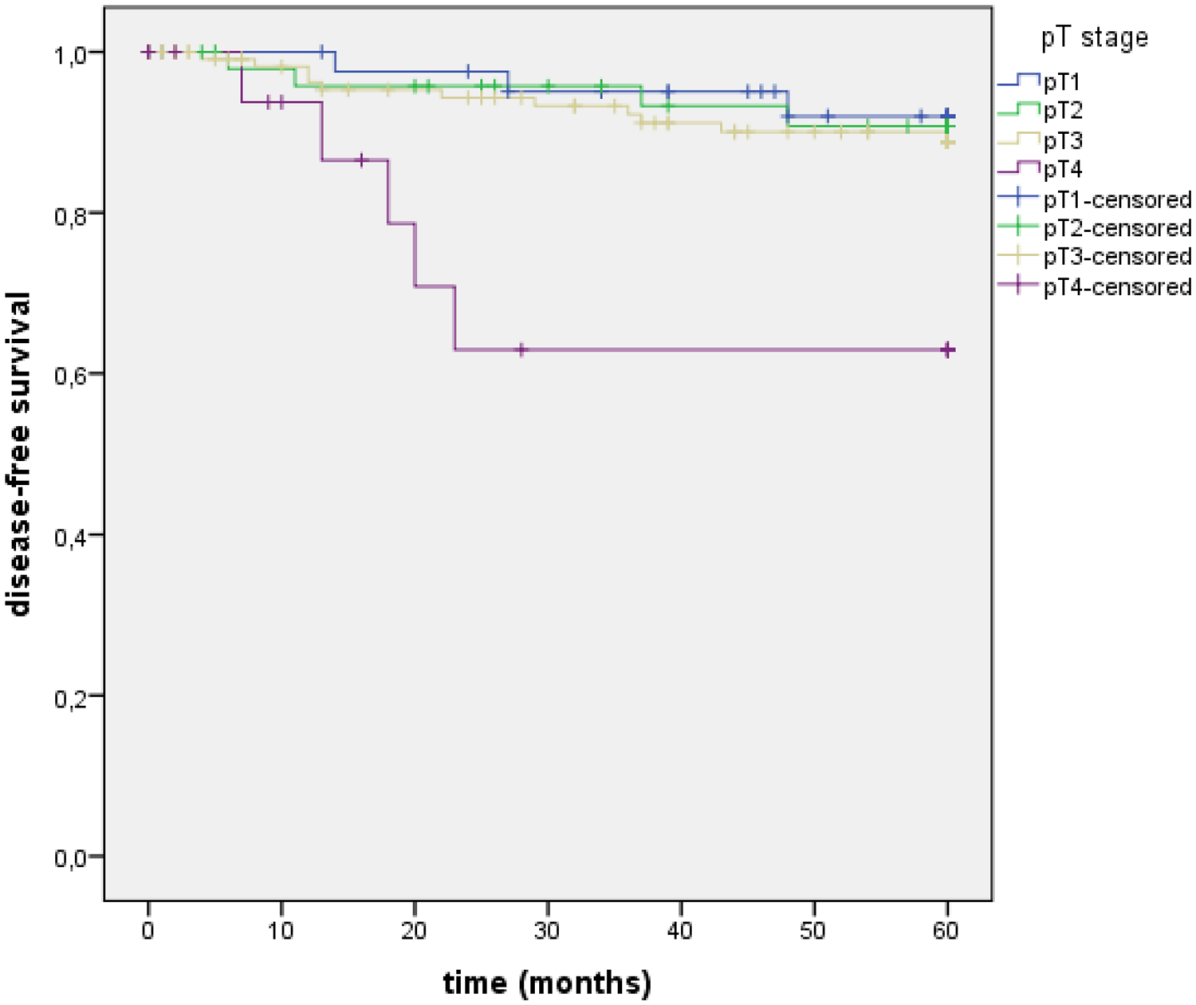
The Kaplan–Meier curves for DFS depending on the pT stage. DFS, disease-free survival

Recurrence was observed in 23 patients (9.9%) and a metachronous colon cancer in four patients (1.7%). In stage UICC I, seven of the 93 patients (7.5%) had a recurrence, and in UICC II, 16 of the 139 patients (11.5%). Depending on the pT-stage, the rate for recurrence was 6.8% (3/44) in pT1, 8.2% (4/49) in pT2, 9.2% (11/120) in pT3, and 26.3% (5/19) in pT4. The median time to recurrence was 19 months (range 4–60, *n* = 22 (*n* = 1 not documented)) with *n* = 16 (69.6%) within the first 36 months. In stage UICC I, the median time to recurrence was 27 months (range 6–48), and in UICC II, 18 months (range 4–60). The disease-free survival (DFS) after 5 years for all patients was 90.1%, for UICC I 92.5%, and for UICC II 88.5% (*p* = 0.206). Figure 2 shows the Kaplan–Meier curves for DFS depending on the pT stage.

The most frequent localization of recurrence was liver (*n* = 15), followed by lungs (*n* = 7), peritoneal (*n* = 4), locoregional (*n* = 2), and others (bones, psoas muscle, and laparotomy scar). Fourteen patients had metastases in a single organ, and nine patients had metastases in multiple organs, mostly the liver and lungs. Recurrent disease was detected in most of the cases in a routine examination (*n* = 16, 69.6%) without symptoms. All seven cases of recurrence in UICC I were detected in a routine examination without symptoms. In four cases, the patient presented during follow-up unscheduled with symptoms (pain, ileus, haematochezia, palpable tumor). In 87.0% (*n* = 20), computed tomography (CT) showed pathological changes, and in 69.6% (*n* = 16), CEA was elevated. Seventy percent (*n* = 14) of patients with a pathological CT had an elevated CEA, and 30.0% (*n* = 6) of the patients had a physiological CEA. The routine follow-up colonoscopy detected three of the four metachronous colon cancer cases after a median time of 38 months (range 15–60). The fourth patient with a metachronous colon primary presented with ileus symptoms. Of the 23 cases with recurrent disease, seven had a history of a tumor before (rectal cancer *n* = 3, breast cancer *n* = 1, prostate cancer *n* = 1, bladder cancer *n* = 1, vulvar carcinoma *n* = 1). Adjuvant chemotherapy was recommended but denied by the patient in four cases with a recurrence. In another patient, liver metastases were initially misdiagnosed as haemangioma. Only ten patients with recurrent disease had no risk factors. The pT-status of these ten patients were pT1 (*n* = 2), pT2 (*n* = 1), and pT3 (*n* = 7), respectively. Patients aged over 80 years had a proportion of 30.4% in the group with recurrence and a proportion of 31.1% in the group without a recurrence. Considering patients with a recurrent disease, the postoperative course after resection of the primary tumor passed without any complications in 52.2% (*n* = 12). Major complications occurred in 26.0% (Clavien-Dindo 3a *n* = 1, 3b *n* = 3, 4 *n* = 2). Table 4 shows baseline information of patients with and without a recurrent disease as well as information of recurrence detection.

**Table 4:** Baseline information of patients with and without a recurrence as well as information of recurrence detection. *UICC*, Union internationale contre le cancer; *ASA*, American Society of Anesthesiologists; *CEA*, carcinoembryonic antigen; *CT*, computed tomography

	**Recurrence *n* = 23**		**No recurrence *n* = 209**	
	UICC I *n* = 7 (30.4%)	UICC II *n* = 16 (69.6%)	UICC I *n* = 86 (41.1%)	UICC II *n* = 123 (58.9%)
**Gender**				
** Male**	4 (57.1%)	8 (50.0%)	48 (55.8%)	62 (50.4%)
** Female**	3 (42.9%)	8 (50.0%)	38 (44.2%)	61 (49.6%)
**Age median (range)**	69.0 (55–82)	75.0 (38–92)	69.5 (35–93)	75.0 (36–94)
**Age ≥ 80 years**	1 (14.3%)	6 (37.5%)	17 (19.8%)	48 (39.0%)
**ASA**				
** I**	1 (14.3%)	0	6 (7.0%)	8 (6.5%)
** II**	3 (42.9%)	7 (43.8%)	43 (50.0%)	53 (43.1%)
** III**	3 (42.9%)	8 (50.0%)	33 (38.4%)	43 (35.0%)
** IV**	0	0	1 (1.2%)	5 (4.1%)
** Not documented**	0	1 (6.3%)	3 (3.5%)	14 (11.4%)
**pT**				
** 1**	3 (42.9%)	0	41 (47.7%)	0
** 2**	4 (57.1%)	0	45 (52.3%)	0
** 3**	0	11 (68.8%)	0	109 (88.6%)
** 4**	0	5 (31.3%)	0	14 (11.4%)
**Grading**				
** I**	1 (14.3%)	0	6 (7.0%)	4 (3.3%)
** II**	5 (71.4%)	15 (93.8%)	69 (80.2%)	99 (80.5%)
** III**	1 (14.3%)	1 (6.3%)	8 (9.3%)	19 (15.4%)
** IV**	0	0	2 (2.3%)	0
** Not documented**	0	0	1 (1.2%)	1 (0.8%)
**Tumor history**				
** Yes**	2 (28.6%)	5 (31.3%)	9 (10.5%)	8 (6.5%)
** No**	5 (71.4%)	11 (68.8%)	77 (89.5%)	115 (93.5%)
**Metastases**				
** Liver**	4 (57.1%)	11 (68.8%)	x	x
** Lungs**	3 (42.9%)	4 (25.0%)	x	x
** Peritoneal**	1 (14.3%)	3 (18.8%)	x	x
** Locoregional**	0	2 (12.5%)	x	x
**Recurrence detection**				
** Detection in routine examination**	7 (100%)	9 (56.2%)	x	x
** CT pathological**	6 (85.7%)	14 (87.5%)	x	x
** CEA elevated**	5 (71.4%)	11 (68.8%)	x	x
** metachronous colorectal primary**	0	0	1 (1.2%)	3 (2.4%)

The therapy of recurrent disease was intended to be curative (*n* = 11/23) in 57.1% (*n* = 4) in stage UICC I and in 43.8% (*n* = 7) in UICC II. In these cases, operative resection, radiofrequency ablation, or radiation was used to treat liver or lung metastases. For seven cases, recurrence occurred only once, and the patients were still alive at the last check-up. In one patient, even multiple recurrences could be treated successfully, being still alive, too. Three patients suffered from progressive disease after the first curative treatment and died subsequently. For the other twelve cases with initial recurrence (*n* = 12/23), palliative chemotherapy was decided, mostly because of a reduced general condition and older age. Only in two patients, the main reason for palliative treatment was an advanced tumor stage without the perspective of curative treatment options. Four patients refused further specific treatment. In one of seven patients over 80 years (14.3%), the therapy was intended to be curative. Two out of four patients with metachronous colon cancer underwent resection in time for a curative attempt; the other two had an advanced tumor stage resulting in a (sub)ileus. Of all patients, 17.7% (*n* = 41/232) died after a median time of 25 months (0–58, *n* = 40 (*n* = 1 not documented)), during the 5-year follow-up.

## Discussion

This study gives an overview of adherence to an intensive follow-up and the corresponding oncological outcome in patients with colon cancer in the early UICC stages I and II.

A postoperative surveillance protocol’s primary goal is to detect a recurrence in an early and resectable stage. Common practice is an intensive 5-year follow-up even for early tumor stages. In recent years, various studies have shown that an intensive follow-up may detect a recurrent disease earlier, however, without improving the survival outcome [5–7, 9]. Next to the aims, such as detection of recurrence and postoperative controls, it must be considered that a surveillance protocol might have negative aspects, too. The frequent examinations are associated with radiation exposure and high costs. The different investigations including colonoscopy can lead to physical stress, especially in elderly patients [10]. On the other side, the surveillance can have a positive psychological impact by reassurance and support, but some patients may also experience anxiety and mental stress [9, 11]. This leads to the question if an intensive follow-up is reasonable in early tumor stages with a low risk of recurrence or for older patients, who will not endure further adjuvant or surgical therapy [12]. Indeed, in our study, the recurrence rate in UICC I was as low as 7.5%, and the recurrence therapy was intended to be curative in only one of seven patients over 80 years (14.3%). Additionally, many patients were lost to follow-up. The reasons for this remain speculative, conceivable are surveillance at another institution, burden, or unconcern.

The risk of recurrence is comparatively low in early tumor stages and increases with higher stages [8, 13]. Patients with a T1N0-tumor have the highest probability of cure with over 90% [2]. In our study, 7.5% of the patients in UICC stage I and 11.5% in UICC stage II had a recurrent disease after a 5-year follow-up. In the stages pT1-3, the recurrence rate was similar with under 10% and increased up to 26.3% in the stage pT4. Other studies confirm this low risk of recurrence in early tumor stages and the increase with higher stages: in the study of Tsikitis et al., 537 patients with early-stage (stages I and IIA) and 254 patients with a late-stage (stages IIB and III) colon cancer were included. The recurrence rate in early-stage disease was as low as 10% compared to over 30% in late-stage disease [8]. Osterman et al. calculated the recurrence risk of 1416 patients with an operated colon cancer. The 5-year recurrence risk was 10% in stage II and considerably higher with 31% in III [14]. The results show that the risk of a recurrent disease considerably differs between the tumor stages and should be noted in the surveillance protocol. Therefore, we suggest that a less intensive surveillance protocol is reasonable for patients in early UICC stages with a low risk of recurrence. For example, the 3-month interval in the first 2 years could be extended to 6 months in UICC stage I, especially for patients with a tumor pT1.

Most surveillance protocols include a CT scan annually and every 3-month CEA measurement as well as clinical examinations. Notably, all patients with recurrences in UICC stage I and most of UICC stage II were detected without any symptoms and abnormalities in the clinical examination in our study. CEA is an antigen, which is produced by epithelial tumor cells in the gastrointestinal tract. Postoperative elevated CEA is associated with recurrent disease. However, in our cohort, CEA was not elevated in approximately one-third of the recurrence, even with conspicuous findings in CT. Different studies describe the insufficient sensitivity and specificity of CEA as a single marker and recommend an interpretation only in combination with other diagnostics [15, 16]. Due to the limited informative value of clinical examination and CEA as a single marker to detect an early recurrence, the regular 3-month visits should be discussed in early tumor stages. However, the measurement of CEA is simple and cheap. Therefore, a family practitioner can perform it easily in patients who wish only a rough surveillance. In our follow-up, pathological alterations were seen by CT scan in 87% of recurrent diseases. The CT has good sensitivity and specificity for detecting early asymptomatic distant recurrence. It is crucial in the postoperative surveillance, but a frequent performance should be avoided due to radiation exposure.

A regular colonoscopy is recommended in the surveillance protocols to detect an anastomotic recurrence or metachronous cancer as patients with colorectal cancer have a higher risk for further colorectal neoplasm [4]. In the study of Ramphal et al., the incidence of metachronous cancer or anastomotic recurrence was 3.1% in the first 6–18 months after curative resection for colorectal cancer. They recommend the first colonoscopy 1 year after the operation [17]. In our study, the rate of metachronous colorectal cancer was as low as 1.7%. Three of the four metachronous colon cancers were detected by a scheduled colonoscopy within the surveillance program, with the earliest 15 months and the latest 60 months postoperative. A colonoscopy is essential in addition to CT scans for early detection of metachronous colorectal cancer. However, because of the low incidence, an extension of the intervals and adjustment to the recommendations of standard colorectal screening could be discussed.

About 70% of the recurrences occurred within the first 3 years in our cohort. These data are in line with other studies and are displayed in surveillance protocols, which recommend less regular examinations after the first 36 months [3, 18]. The median time to recurrence tends to be shorter in patients with advanced disease compared to patients with an early-stage disease [8, 18]. In UICC I, the median time was 27 months, and in UICC II, it was 18 months in our study. Despite a longer time to recurrent disease, we detected no recurrence after 48 months in patients with an UICC stage I. Consequently, not only a less intensive surveillance protocol but also a shortened one could be discussed for patients in UICC stage I. As patients with a pT4-tumor had a higher risk of recurrence with 26.3% compared to about 10% in pT1-3, we suggest the whole 5-year surveillance at least for patients with pT4.

The cure of patients in tumor stages UICC I or II is considerably higher than in more advanced tumor stages [2]. In our study, the therapy of nearly two-thirds of the patients in UICC I with recurrent disease was intended to be curative. The most common reasons for palliative treatment were reduced general condition and older age. Berg et al. showed that age over 70 years at diagnosis is a poor prognostic factor for cancer-specific survival [2]. Tran et al. described a high rate for hospitalization after endoscopy for older patients, demonstrating how stressful the surveillance protocol, including the colonoscopy, might be for elderly patients [10]. Therefore, it should be discussed with patients in a reduced general condition or older age if a full intensive surveillance protocol and its consequences are reasonable. Defining elderly patients in terms of years is difficult, and the age is definitely not the only parameter. Next to the chronological age, fitness and biological age must be considered. We propose the age of 80 years as a benchmark since most patients with this age usually present with some kind of frailty limitating the options for a curative multimodal treatment of a tumor recurrence. However, in elderly patients, the decision if a surveillance protocol is performed or not is always an individual one, based on general condition and patient’s wish.

There is an ongoing discussion if all patients in every tumor stage should undergo the same intensive follow-up program. By reducing the frequency, metastases may be detected later, possibly resulting in an advanced tumor stage with a worse overall survival. However, a later diagnosis of recurrence does not automatically implicate palliative treatment, since most patients nowadays are able to undergo markedly improved curative multimodal treatment options such as chemotherapy, immunotherapy, liver resection, liver radiofrequency ablation, and lung radiotherapy. The rather high drop-out rate during regular follow-up can be interpreted as an argument for a less intensive follow-up or even renouncement of follow-up. Finally, recent studies described no difference in the survival after intensive and less intensive surveillance [5–7].

In contrast to other guidelines, the “National Comprehensive Cancer Network (NCCN)” recommends, for patients in stage I, colonoscopy only and no further specific surveillance. The risk of recurrence, especially in tumor stage UICC I, is low, as in our study with less than 10% after a 5-year follow-up. In over 90% of the patients, no recurrence was detected, but an intensive follow-up with regular clinical visits, blood analyses, CT scans, and colonoscopies was recommended. Therefore, we would suggest that a less intensive follow-up in patients with an early tumor stage, especially UICC I, is reasonable. The 3-month interval could be extended to 6 months, even in the first 2 years. Likewise, a CT scan might be performed only after 12 and 36 months. The whole surveillance time could be shortened for patients in UICC stage I (e.g., 3 years). With patients in a bad general condition or advanced age who probably will not undergo a specific therapy for recurrence, it is to discuss if surveillance should be performed at all.

Our analysis has some limitations. The number of included patients is small, describing tendencies but no significance. The results and suggested changes for the surveillance protocol must be confirmed in further studies with a large cohort. Due to the retrospective design, the results depend on the documented data quality. We had a considerable drop-out rate of the follow-up with 44.8% after a median time of less than a year. For most of the patients, the reason for dropping-out is unknown. A follow-up in another hospital is not unlikely because there are a few hospitals in the region. However, most patients live in the city, and the university hospital is the only public hospital there. Additionally, in this region, patients have a strong connection to their family doctor, implicating that the patients maybe have non-specific follow-up examinations there. As we do not know if the patients lost to our follow-up had a recurrent disease or not, the recurrence rate in summery could be higher or even lower. As the lost patients have not received therapy for recurrence at our hospital, we suggest that no recurrent disease was detected and, consequently, that the recurrence rate is significantly lower. Finally, the high drop-out rate during follow-up could be interpreted as additional argument for a reduced or shortened surveillance, since many patients anyway do not adhere to the appointments.

## Conclusion

In conclusion, the risk of recurrence is low in patients with localized colon cancer, especially in UICC stage I. We suggest that a less intensive surveillance protocol (e.g., prolongation of intervals, reduction of examinations, shortening of the follow-up length) for these patients is reasonable. Additionally, the follow-up rationale should be discussed in elderly patients, who are frail or do not qualify for curative or palliative treatment options.

## Data Availability

The data underlying this article will be shared upon reasonable request to the corresponding author.
